# Replicative DNA polymerase mutations in cancer^☆^

**DOI:** 10.1016/j.gde.2013.12.005

**Published:** 2014-02

**Authors:** Ellen Heitzer, Ian Tomlinson

**Affiliations:** 1 Institute of Human Genetics, Medical University of Graz, Harrachgasse 21/8, A-8010 Graz, Austria; 2 Molecular and Population Genetics Laboratory, Wellcome Trust Centre for Human Genetics, Roosevelt Drive, Oxford OX3 7BN, UK; 3 Oxford NIHR Comprehensive Biomedical Research Centre, Wellcome Trust Centre for Human Genetics, Roosevelt Drive, Oxford OX3 7BN, UK

## Abstract

Three DNA polymerases — Pol α, Pol δ and Pol ɛ — are essential for DNA replication. After initiation of DNA synthesis by Pol α, Pol δ or Pol ɛ take over on the lagging and leading strand respectively. Pol δ and Pol ɛ perform the bulk of replication with very high fidelity, which is ensured by Watson–Crick base pairing and 3′exonuclease (proofreading) activity. Yeast models have shown that mutations in the exonuclease domain of Pol δ and Pol ɛ homologues can cause a mutator phenotype. Recently, we identified germline exonuclease domain mutations (EDMs) in human *POLD1* and *POLE* that predispose to ‘polymerase proofreading associated polyposis’ (PPAP), a disease characterised by multiple colorectal adenomas and carcinoma, with high penetrance and dominant inheritance. Moreover, somatic EDMs in *POLE* have also been found in sporadic colorectal and endometrial cancers. Tumors with EDMs are microsatellite stable and show an ‘ultramutator’ phenotype, with a dramatic increase in base substitutions.

**Current Opinion in Genetics & Development** 2014, **24**:107–113

This review comes from a themed issue on **Cancer genomics**

Edited by **David J Adams** and **Ultan McDermott**

For a complete overview see the Issue and the Editorial

Available online 26th February 2014

0959-437X/$ – see front matter, © 2014 The Authors. Published by Elsevier Ltd. All rights reserved.

**http://dx.doi.org/10.1016/j.gde.2013.12.005**

## Introduction

DNA polymerases are responsible for synthesis of DNA and are essential for replication, DNA repair and genetic recombination. DNA replication is a highly complex process and in eukaryotes it involves multiple enzymes including the B family polymerases Pol α, Pol δ, and Pol ɛ [1,2]. These enzymes catalyse the polymerisation of deoxyribonucleotides into the nascent DNA strand. While Pol α initiates DNA synthesis, Pol δ and Pol ɛ replace Pol α after primer extension and perform the bulk of DNA replication. Most polymerases lack intrinsic error-checking activity, relying on Watson–Crick base pairing for their fidelity. However, the proofreading (exonuclease) domains of Pol δ and Pol ɛ ensure that these polymerases have a particularly low error rate, of the order of 10^−7^ substitution mutations per base. A variety of *in vitro* studies has shown that proofreading improves replication fidelity approximately 100-fold [3^•^,4].

The Pol δ and Pol ɛ enzymes are heterotetramers in higher eukaryotes. Both Pol δ and Pol ɛ comprise a catalytic subunit, POLD1 and POLE respectively, and accessory subunits (POLD2/3/4 and POLE2/3/4) that interact with cofactors such as Proliferating Cell Nuclear Antigen (PCNA) [5]. Both genes are ubiquitously expressed and show high levels of evolutionary conservation. The two polymerases differ from each other throughout most of their length, but are homologous (23% identity, 37% similarity) over their exonuclease domains (residues 268–471 of POLE and 304–517 of POLD1).

Based on studies in yeast, it has been shown that Pol δ and Pol ɛ usually replicate the leading and lagging strand respectively [6,7^•^]. However, it is still not fully elucidated whether this is always the case at replication forks. Pavlov proposed a model where Pol ɛ starts replicating the leading strand, but may later dissociate, and Pol δ then takes over to complete the replication [8]. A higher mutation rate in Pol δ exonuclease deficient yeast strains compared to Pol ɛ exonuclease-deficient strains endorses this hypothesis [8–10].

There is substantial evidence that in addition to DNA synthesis, Pol ɛ and Pol δ play essential roles in repair of chromosomal DNA [8,11,12]. Pol ɛ and Pol δ are thought to be involved in several repair pathway including nucleotide excision repair (NER), ismatch repair (MMR) and repair of double strand breaks (DSBR) [12,13].

## Polymerase proofreading defects cause mutator phenotypes

Replication fidelity has been extensively studied in yeast and other microbes, though less is known about the impact of proofreading-defective DNA polymerase mutations in higher eukaryotes. The exonuclease domain catalyses the preferential hydrolysis of non-complementary nucleotides at the 3′-terminus, and in yeast, inactivating missense EDMs of Pol ɛ and Pol δ cause a base substitution mutator phenotype with variable severity [9,10,14–17]. It has been suggested that in yeast, Pol ɛ and Pol δ proofread opposite strands at defined replication origins and may proofread for each other [6,18,19]. Data from mice with homozygous germline *Pole* and/or *Pold1* mutations at the exonuclease active site were shown to have distinct, but overlapping tissue-specific tumor phenotypes. *Pole*-mutant animals predominantly had nodal lymphomas and histiocytic sarcomas, whereas *Pold1* mutants had thymic lymphomas and skin papillomas/sarcomas. Both types of mice had intestinal adenomas (more in *Pole*) and lung tumors (more in *Pold1*). Double knockout animals died early from thymic lymphoma. Spontaneous mutations frequencies were higher in *Pole* mutants than *Pold1* mutants [20^••^]. One explanation could be that the fidelity of lagging strand replication is greater than that of leading strand, because post-replicative DNA mismatch repair (MMR) preferentially corrects lagging strand replication errors [21,22]. However, this in contrast with the data from yeast [14]. Genetic studies in proofreading-deficient, haploid yeast strains which also carried a MMR-defect showed a synthetically lethal phenotype indicating a synergistic effect on the mutation rate of proofreading and MMR [23,24]. This was also confirmed in mouse studies where loss of both proofreading and MMR led to embryonic lethality [20^••^,25]. Conversely, others have speculated that MMR deficiency may be required for the EDM mutator phenotype to be manifested [26].

## Germline mutations in *POLD1* and *POLE* cause polymerase proofreading-associated polyposis (PPAP)

Even if replication fidelity is high, some errors always escape proofreading and are then corrected by MMR [27]. In studies beginning in the late 1980s, it was found that germline mutations in four MMR genes (*MSH2*, *MLH1*, *MSH6* and *PMS2*) were causative for the hereditary colorectal and other cancers that are present in Lynch syndrome (reviewed in [28,29]). Furthermore, somatic silencing of *MLH1* expression occurs in several cancer types, notably CRC and endometrial cancer (EC). In addition, bi-allelic germline *MUTYH* mutations predispose to adenomatous colorectal polyposis and CRC through defective base excision repair. We recently identified specific germline EDMs in *POLD1* and *POLE* that are causative for the development of multiple colorectal adenomas and CRC. Since the phenotype overlaps with those who carry germline mutations in *MUTYH* and the MMR genes, we have called the disease PPAP [30,31^••^].

Using a combination of whole-genome sequencing of highly selected multiple adenoma patients, linkage analysis, and studies of loss-of-heterozygosity (LOH) in tumors, followed by replication in a large set of familial CRC cases [31^••^] we identified one germline mutation in *POLE* (p.Leu424Val) and one in *POLD1* (p.Ser478Asn) that were not present in nearly 7000 UK controls or in public databases of controls. In addition, another probably pathogenic mutation, *POLD1* p.Pro327Leu, was found in a further patient with multiple adenomas. Patients who carry EDMs in *POLE* or *POLD1* show variable phenotypes: some have tens of adenomas that do not appear to progress rapidly to cancer, whereas others have a small number of large adenomas or early-onset carcinomas, thus resembling Lynch syndrome. Interestingly, female carriers of *POLD1* p.Ser478Asn have a greatly increased risk of EC. Segregation analysis confirmed a dominant, high-penetrance predisposition to colorectal adenomas. Smith *et al.* have subsequently proposed an additional predisposing *POLE* mutation outside the exonuclease domain [32].

Although there are several single nucleotide polymorphisms (SNPs) located at conserved sites within the polymerase or exonuclease domains of *POLE* and *POLD1*, genome-wide association studies and a few targeted studies have found no associations with cancer risk to date [33–38]. However, a common polymorphism within *POLD3* has been found to be associated with an increased risk of CRC in the general northern European population [39], although the mechanism of action is unknown.

## Somatic mutations in *POLD1* and *POLE*

Until recently, several studies had suggested the presence of pathogenic somatic DNA polymerase mutations in cancer, but these studies were too small to show true functionality, many cancers were MMR-deficient (and hence had a high background mutation rate), and EDMs were not distinguished from other polymerase mutations. The relatively-recent Cancer Genome Atlas (TCGA) exome sequencing project has provided the best evidence for *POLE* being the target of recurrent somatic mutations in MMR-proficient, but ‘ultramutated’ CRCs [40^••^]. Further analysis showed that the mutations causing the ultramutator phenotype were all EDMs [31^••^,40^••^,41]. In the initial TCGA cohort, there were 7 *POLE* non-synonymous EDMs out of a total of 226 CRCs (3%). All of these cancers were microsatellite-stable (i.e. *prima facie* having normal MMR). Although the germline p.Leu424Val change was absent, two recurrent changes were found, p.Val411Leu and p.Ser459Phe. In addition a further recurrent *POLE* EDM, p.Pro286Arg, was found by a different CRC exome sequencing project [42]. No equivalent *POLD1* mutations have been reported for CRC. One possible explanation is that Pol ɛ and Pol δ act independently in different cells and various cancers might have differential mutational hotspots in oncogenes and tumor suppressors that are replicated from different polymerases [43,44].

Due to the fact that *POLD1* germline mutations predispose to EC, we looked for somatic *POLE* and *POLD1* mutations in sporadic ECs. We found *POLE* EDMs in about 7% of cancers, including some previously detected in CRCs and one mutation affecting the exonuclease active site. Similar to CRC, *POLE* mutations in ECs were associated with an ultramutator, but microsatellite-stable phenotype, characterised by an excess of substitution mutations [45^•^]. As for CRC, there were no recurrent *POLD1* EDMs in ECs. TCGA EC project had similar findings [46^•^].

## Mechanisms of polymerase EDM-driven tumorigenesis

Structural data strongly suggest that the *POLE* and *POLD1* EDMs impair polymerase proofreading. Mapping of the reported mutations onto a hybrid structure of yeast DNA polymerase (3iay) and T4 polymerase shows that they mostly lie along the DNA-binding pocket of the exonuclease domain [31^••^]. POLE p.Leu424Val and POLD1 p.Ser478Asn pack together at the interface between two helices that form the base of the exonuclease active site. The most common somatic *POLE* mutation (p.Arg286His) localises to the DNA binding pocket adjacent to the exonuclease active site, probably perturbing the structure of the DNA binding pocket. Data from the equivalent residue mutation, p.Pro123Leu, in T4 bacteriophage that produces a strong mutator phenotype confirm this hypothesis [47]. POLE amino acid 297 interacts with exonuclease active site residue 275, and mutations here would probably alter the active site conformation. POLE residue 411, however, is not predicted to interact with DNA or catalytic site residues, suggesting that the increased mutation rate may result from secondary effects on the binding pocket. Hypermutation is, in summary, a very plausible consequence of *POLE* and *POLD1* EDMs.

**Figure 1:**
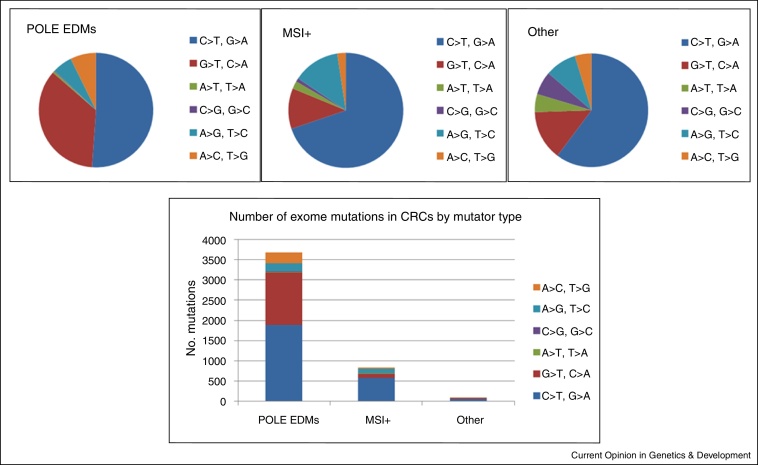
Mutation spectra (upper) and numbers (lower) in exome sequence data from TCGA project colorectal cancers of three types. Note that MSI+ is used synonymously with MMR-deficient.

**Figure 2:**
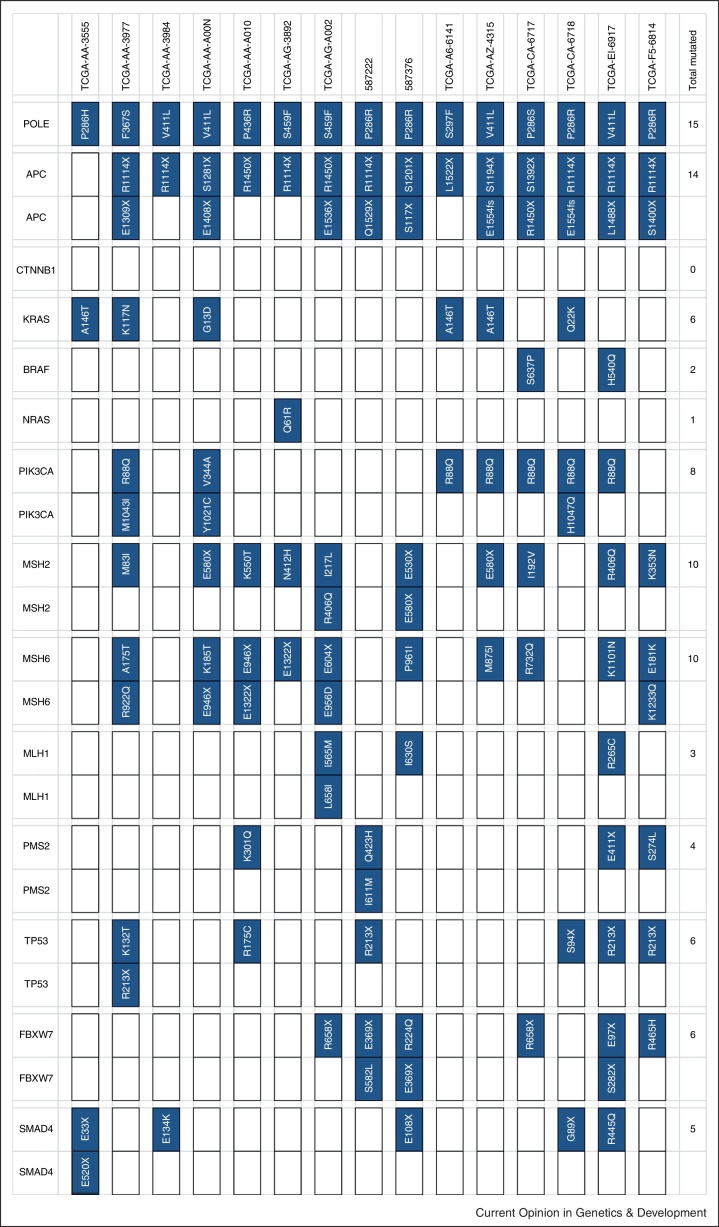
Mutations in colorectal cancer driver genes in the 17 POLE EDM TCGA project colorectal cancers (data from December 2013). Note the following: (i) bi-allelic mutations are shown for putative tumor suppressor genes, and additionally for *PIK3CA*; (ii) some filtering of variants with a low chance of being pathogenic has been performed on a gene-by-gene basis (e.g. for *APC*, only protein-truncating or splice-site mutations were considered pathogenic, for *CTNNB1*, only mutations affecting the exon 3 phosphorylation sites were considered pathogenic, *et cetera*); (iii) some highly atypical but potentially pathogenic mutations may therefore not be shown for some genes; (iv) LOH is not shown; (v) some of the mutations shown are highly likely to be passengers, especially missense changes in genes (e.g. MMR genes, SMAD4) where hotspots are not established; (vi) the presence of two mutations does not necessarily imply that these are bi-allelic changes.

Exome and targeted sequencing data clearly show the mutation spectra of tumors with *POLE* and *POLD1* EDMs [31^••^,40^••^,48]. Compared to *POLE*-wild type tumors, EDM-tumors have an increased tendency for somatic base substitutions of all types, typically with about 5000 substitutions in the coding regions alone (Figure 1). C:G > T:A changes generally remain the most common, but there is a particular increase in the proportion of G:C > T:A and A:T > C:G transversions. Since p.Pro286Arg mutant tumors show a much stronger bias towards transversions than cancers with p.Val411Leu, there is considerable evidence that specific *POLE* mutations have different effects on the somatic mutation spectrum. It is of note that somatic mutations secondary to defective proofreading tend to occur at sites flanked by an A base on the “positive” DNA strand, rather than by T, G or C. The causes for this observation are currently unknown, although lower helix ‘melting’ temperatures of A:T tracts are a plausible contributing factor. Notably, in CRCs with EDMs, the spectrum and/or frequency of known driver mutations is unusual (Figure 2). Recurrent mutations are frequently observed in the known CRC driver genes, but these are often of types and at positions other than the common hotspots. Examples include nonsense changes at codon 1114 of *APC*, 1322 of *MSH6* and 213 of *TP53*, and missense mutations at codons 117 and 146 of *KRAS* and 88 of *PIK3CA* [31^••^,49]. Some of these mutations, such as *KRAS* p.Lys117Asn occur adjacent to oligo(A) tracts and hence at putative hypermutable sites in a proofreading-deficient background. We speculate that such mutations might be functionally suboptimal with respect to the ‘classical’ mutations, such as those at *KRAS* codons 12 and 13, yet are tolerated because the ultramutator cancer can acquire additional, advantageous mutations rapidly; we have termed this the ‘mini-driver’ or ‘polygenic’ model of tumorigenesis. However, other recurrent mutations, such as *PIK3CA* p.Arg88Gln, do not occur in at A:T-rich context. Perhaps these ‘atypical’ *PIK3CA* mutations are more selectively advantageous than classical *PIK3CA* mutations, such as codons 545 or 1047, in the context of *POLE* deficiency. The data also suggest that somatic *POLE* mutations occur very early during colorectal tumorigenesis, because the frameshift mutations found often at *APC* in unselected CRCs are not seen in tumors with EDMs.

*POLE* and *POLD1* may not to act as classical tumor suppressor genes. Enzyme loss-of-function mutations are thought unlikely to be pathogenic, since for proofreading can fail, successful polymerisation must have occurred first. Another point against a classical tumor suppressor model is the fact that only a minority of tumors with *POLE* or *POLD1* EDMs show LOH or other inactivating mutations that could act as ‘second hits’. On the other hand, data from mice only indicate a mutator phenotype and increased frequency of tumor formation when *Pole* mutations are homozygous [20^••^]. Overall, we can certainly envisage a situation in which the pathogenic EDMs are selectively haploinsufficient, but we also note that somatic *MSH2* and *MSH6* mutations secondary to the EDM are common (Figure 2) and may contribute to tumorigenesis.

## Perspectives

Although mutations in the exonuclease domain of *POLD1* and *POLE* have previously been described in yeast and mouse models, the identification of germline and somatic mutations that drive tumorigenesis in humans is a recent finding. However, the consequences of polymerase EDMs are not yet clear and further analysis will be needed to understand how these mutations contribute to tumorigenesis. We do not know how proofreading fails or why the resulting mismatch is not repaired by either a wildtype copy of POLE or POLD1 or by MMR. There is additionally intriguing speculation that patients with *POLE*-mutant CRCs and ECs have superior survival to those with other patients, perhaps as a result of the general or specific mutation burden conferred by the ultramutator phenotype. That same burden might also make those ultramutator cancers sensitive to mutation-inducing or DNA repair-blocking therapies. Finally, we emphasise that although pathogenic polymerase EDM cancers form a rare subtype of tumor apparently restricted to the colorectum and endometrium, there is no reason to regard them as an unimportant group. On the contrary, fine-scale classification of cancers using molecular and other methods is likely to form the basis of improved patient management in the future.

## References and recommended reading

Papers of particular interest, published within the period of review, have been highlighted as:

• of special interest
•• of outstanding interest
